# Supplementary material for: Identification and Pharmacokinetic Studies on Complanatuside and Its Major Metabolites in Rats by UHPLC-Q-TOF-MS/MS and LC-MS/MS
Source: Molecules. 2018 Dec 25;24(1):71. doi: 10.3390/molecules24010071 (PMC6337141; doi:10.3390/molecules24010071)
Supplement: Supplementary file 1 [file molecules-24-00071-s001.pdf]

Supplementary Material

Contents

The negative MS/MS spectra of the identified 34 metabolites from the sample of rats of complanatuside.

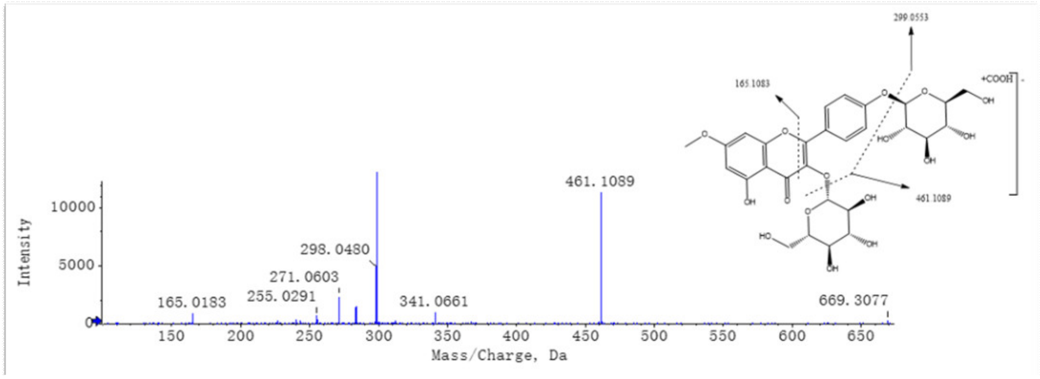

Figure S1 The negative MS/MS spectra of metabolite M1

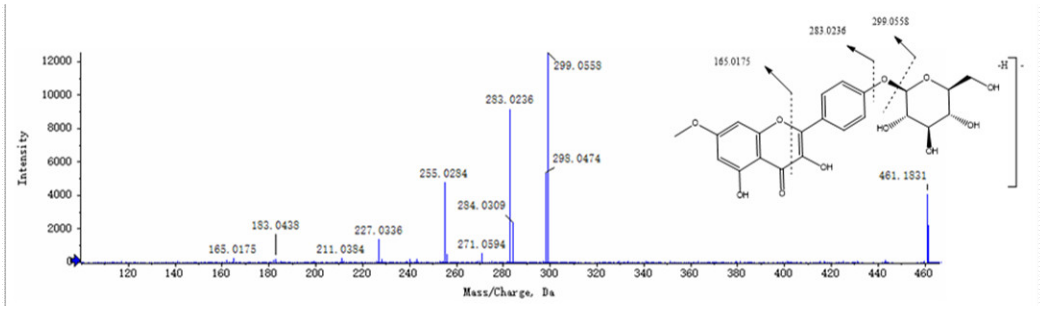

Figure S2 The negative MS/MS spectra of metabolite M2

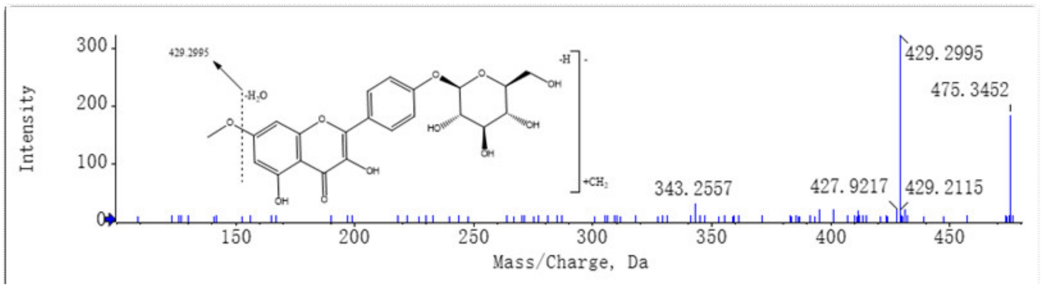

Figure S3 The negative MS/MS spectra of metabolite M3

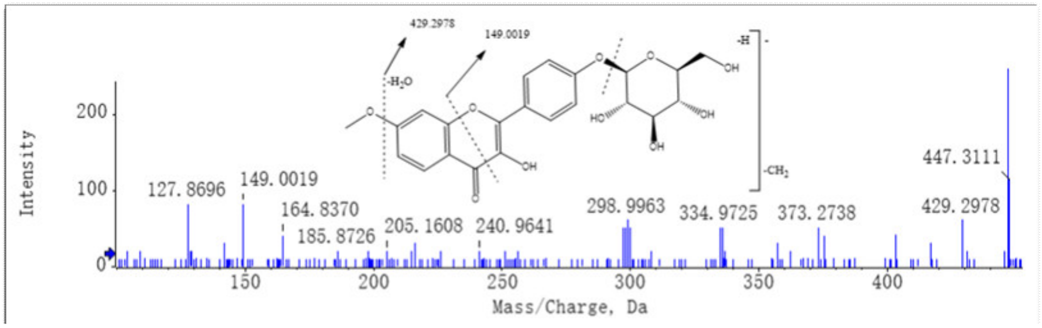

Figure S4 The negative MS/MS spectra of metabolite M4

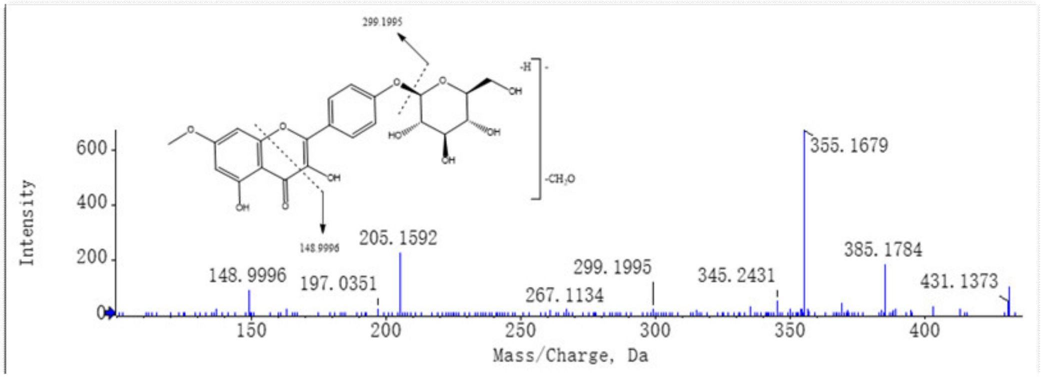

Figure S5 The negative MS/MS spectra of metabolite M5

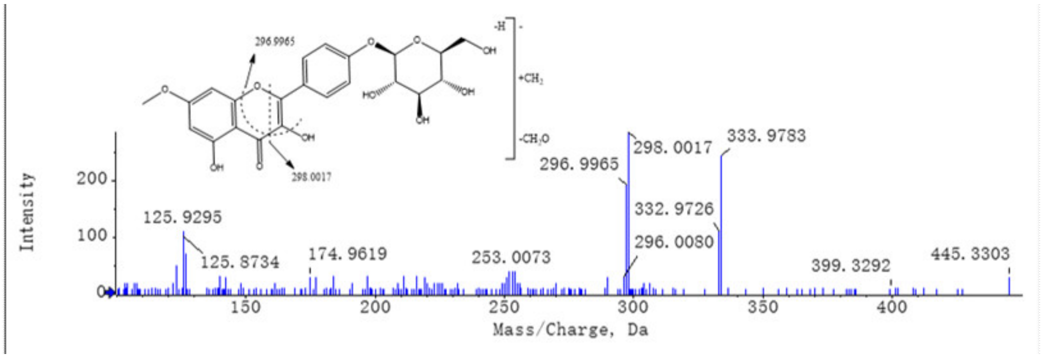

Figure S6 The negative MS/MS spectra of metabolite M6

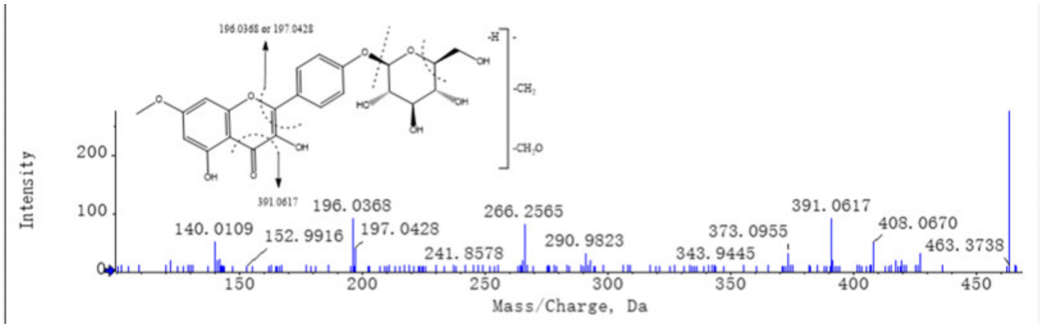

Figure S7 The negative MS/MS spectra of metabolite M7

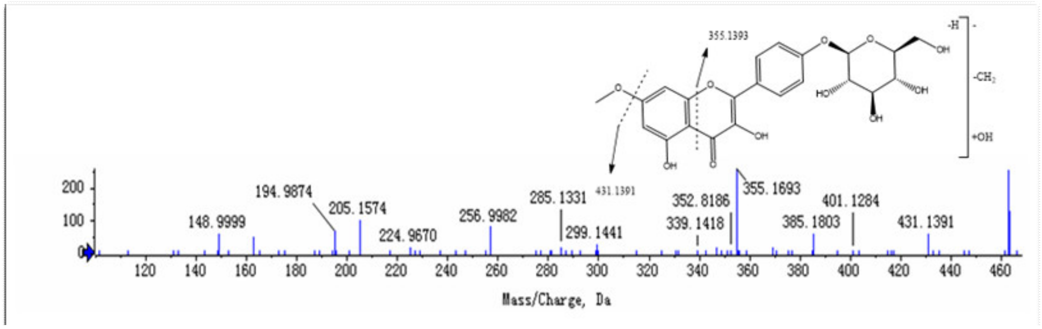

Figure S8 The negative MS/MS spectra of metabolite M8

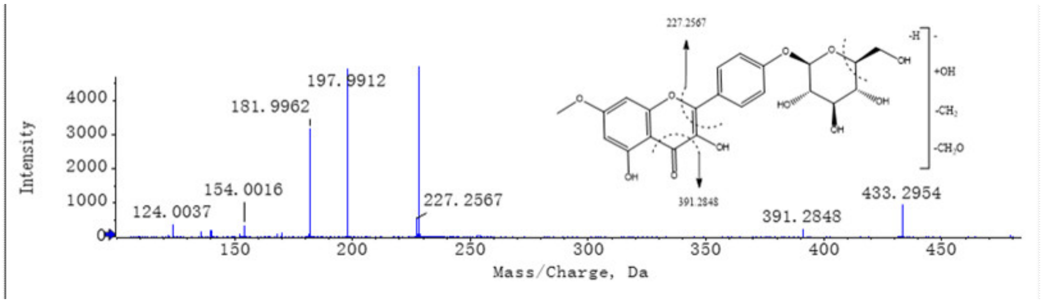

Figure S9 The negative MS/MS spectra of metabolite M9

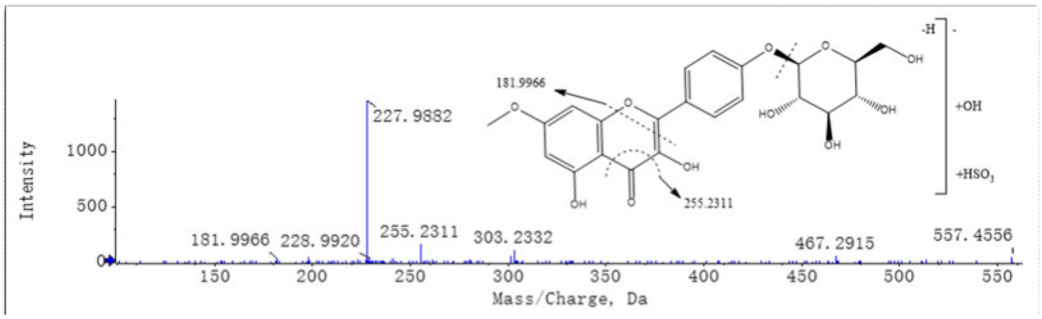

Figure S10 The negative MS/MS spectra of metabolite M10

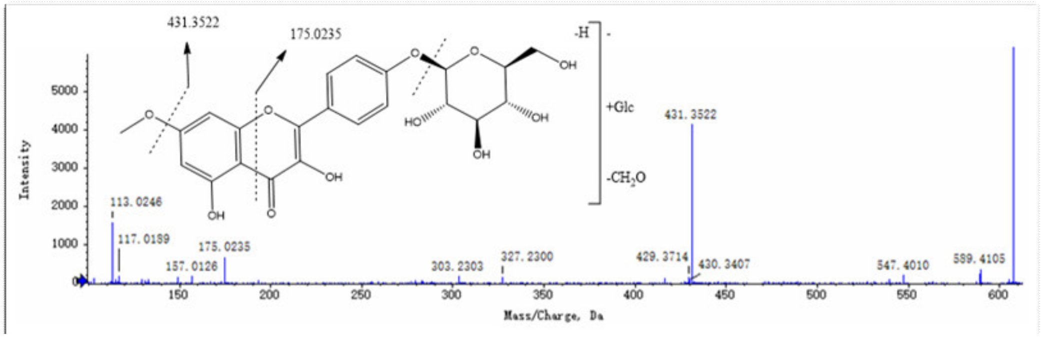

Figure S11 The negative MS/MS spectra of metabolite M11

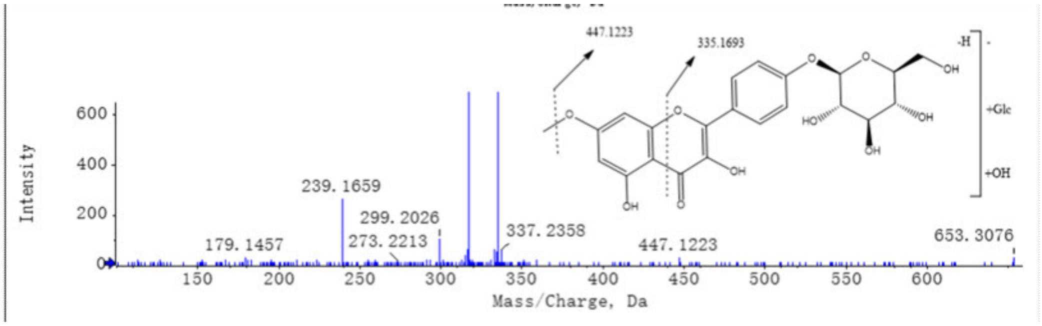

Figure S12 The negative MS/MS spectra of metabolite M12

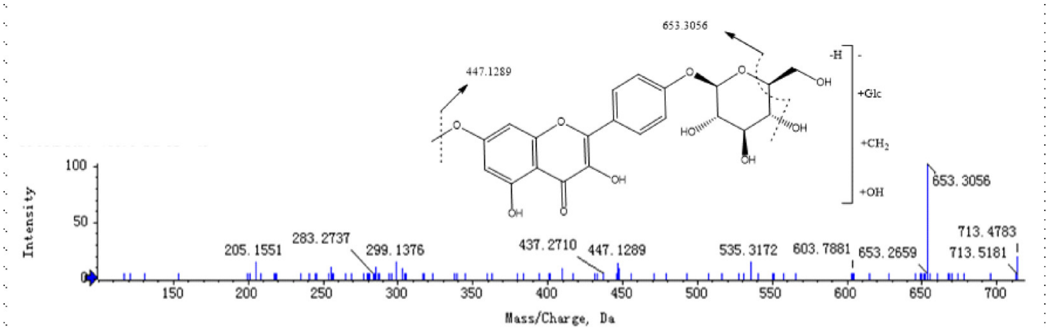

Figure S13 The negative MS/MS spectra of metabolite M13

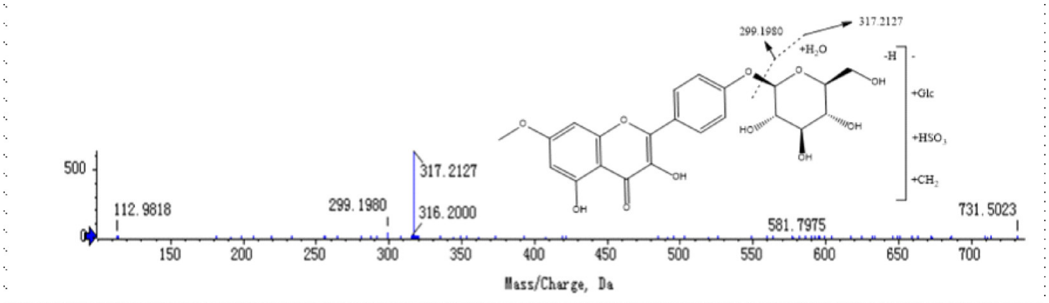

Figure S14 The negative MS/MS spectra of metabolite M14

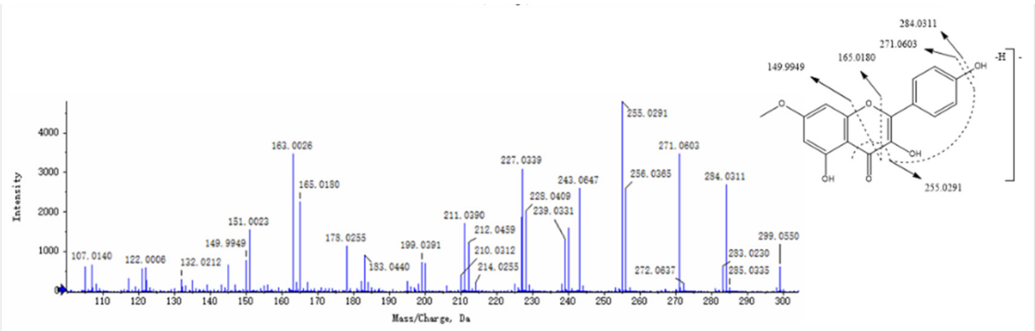

Figure S15 The negative MS/MS spectra of metabolite M15

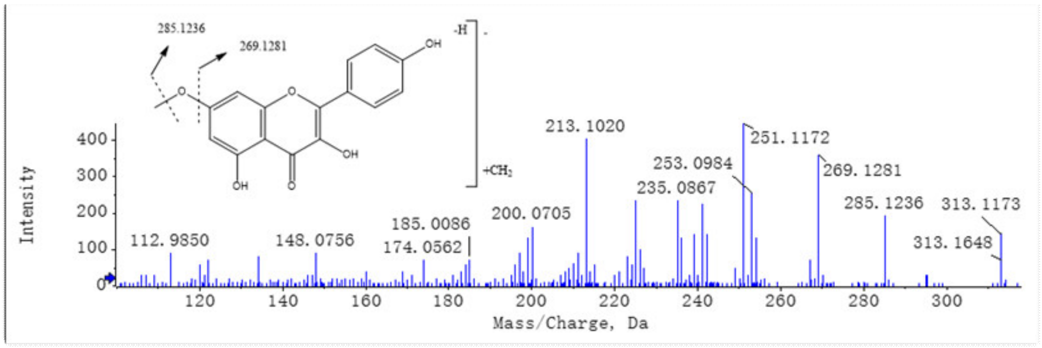

Figure S16 The negative MS/MS spectra of metabolite M16

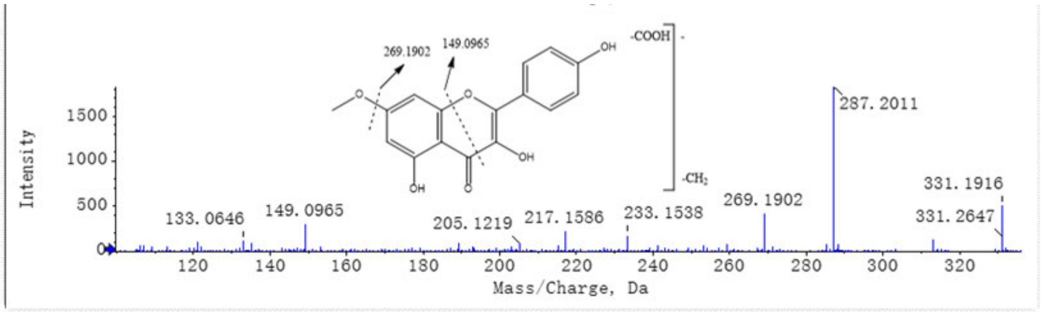

Figure S17 The negative MS/MS spectra of metabolite M17

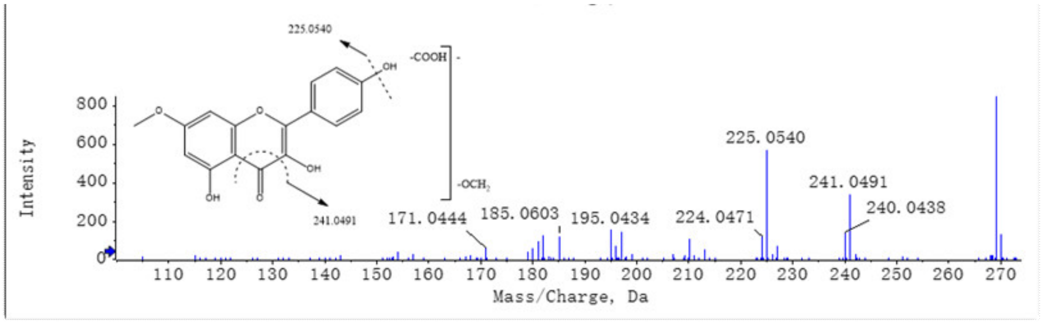

Figure S18 The negative MS/MS spectra of metabolite M18

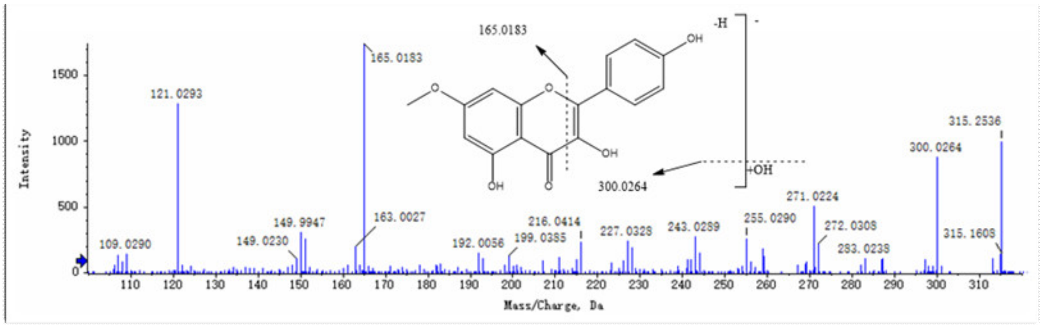

Figure S19 The negative MS/MS spectra of metabolite M19

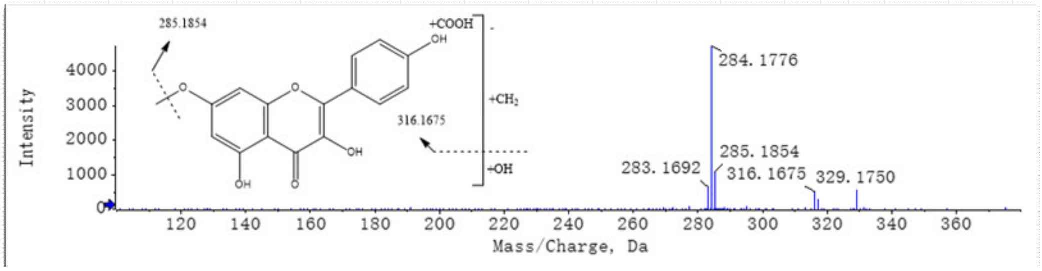

Figure S20 The negative MS/MS spectra of metabolite M20

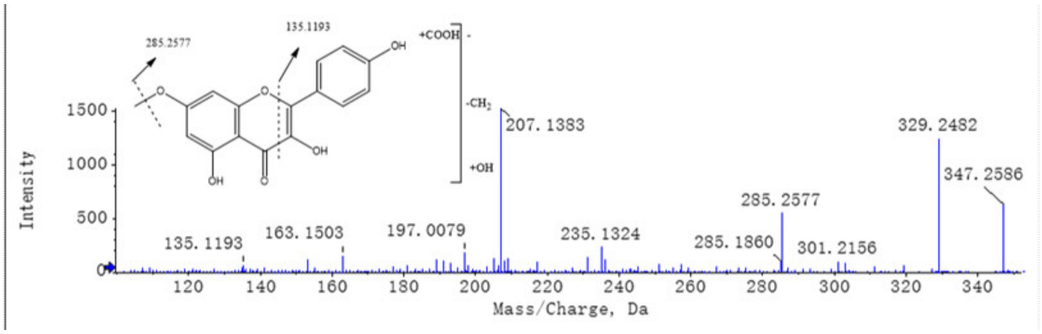

Figure S21 The negative MS/MS spectra of metabolite M21

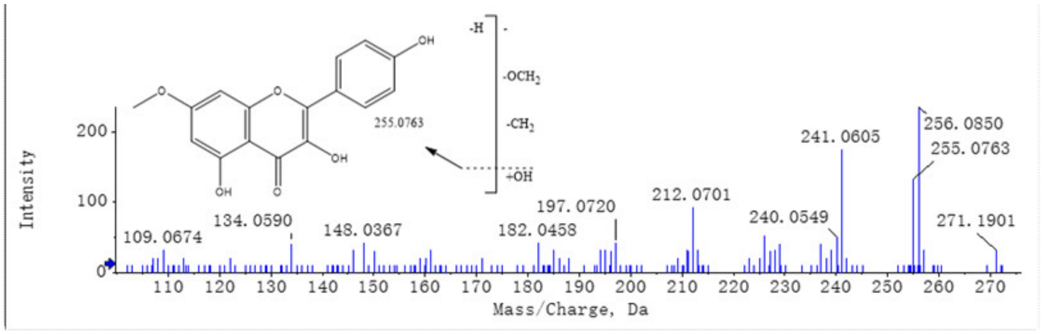

Figure S22 The negative MS/MS spectra of metabolite M22

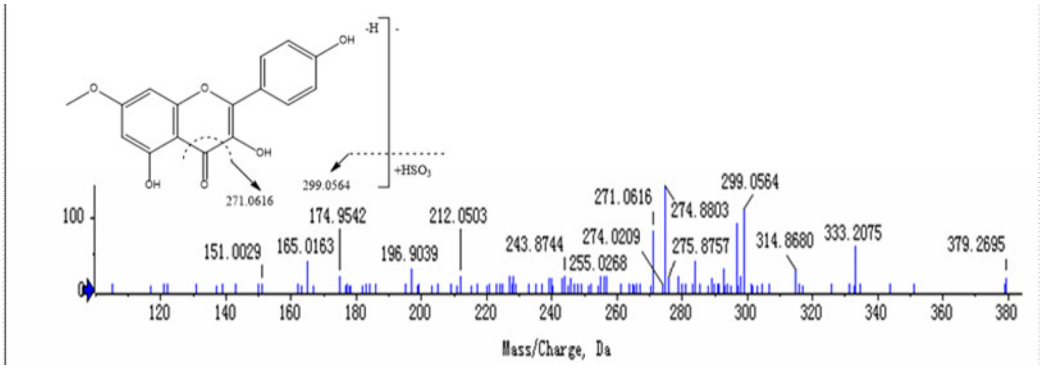

Figure S23 The negative MS/MS spectra of metabolite M23

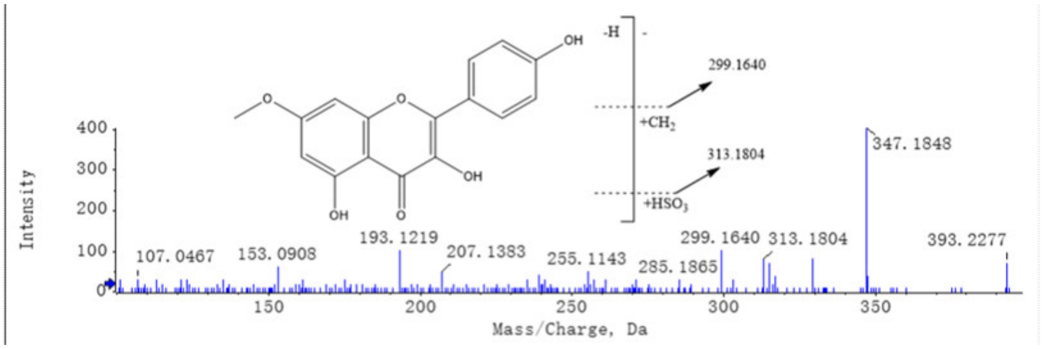

Figure S24 The negative MS/MS spectra of metabolite M24

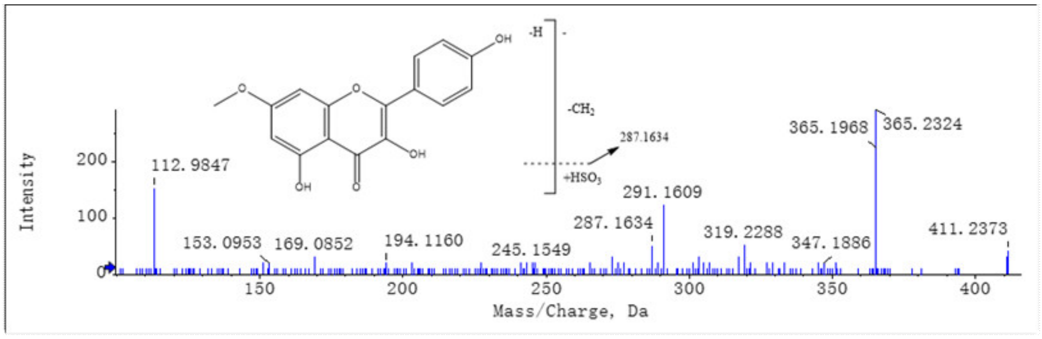

Figure S25 The negative MS/MS spectra of metabolite M25

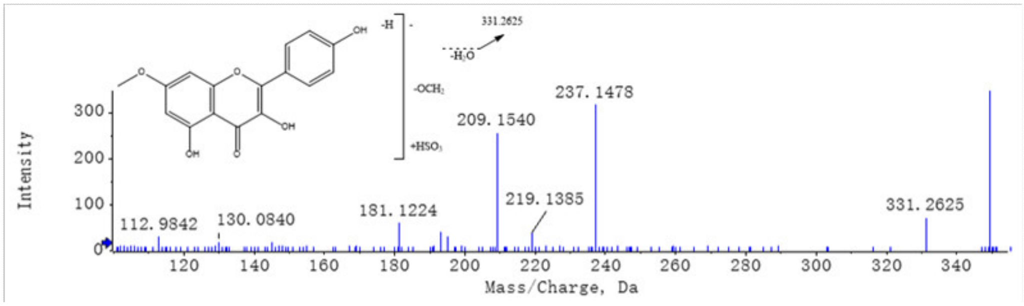

Figure S26 The negative MS/MS spectra of metabolite M26

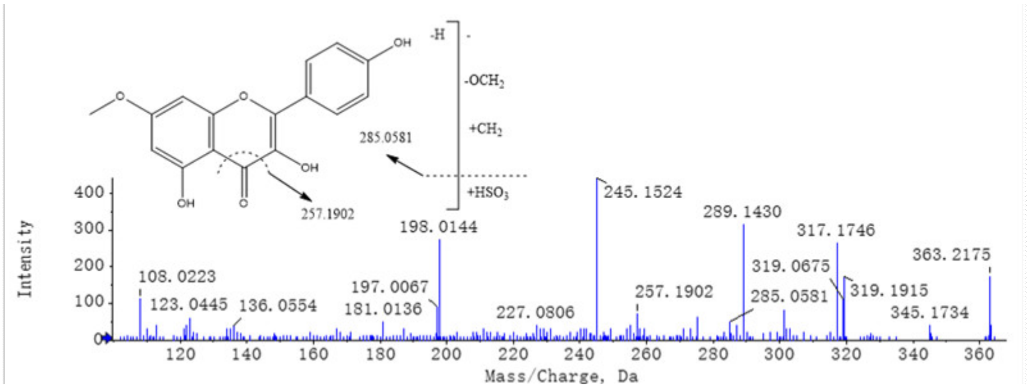

Figure S27 The negative MS/MS spectra of metabolite M27

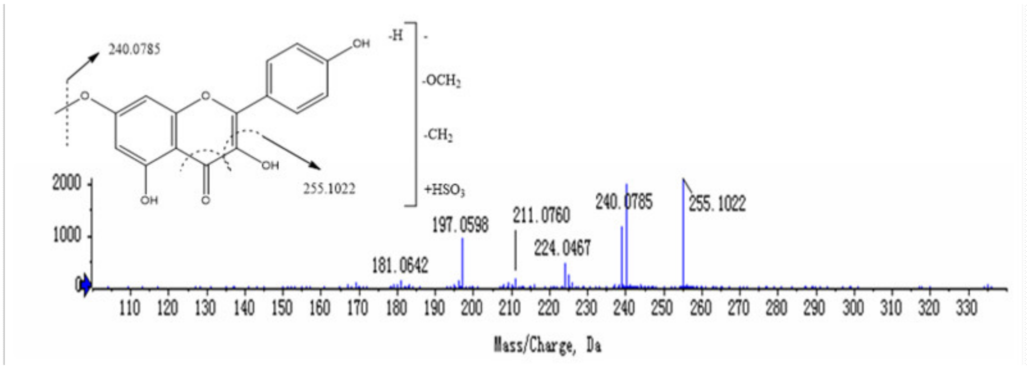

Figure S28 The negative MS/MS spectra of metabolite M28

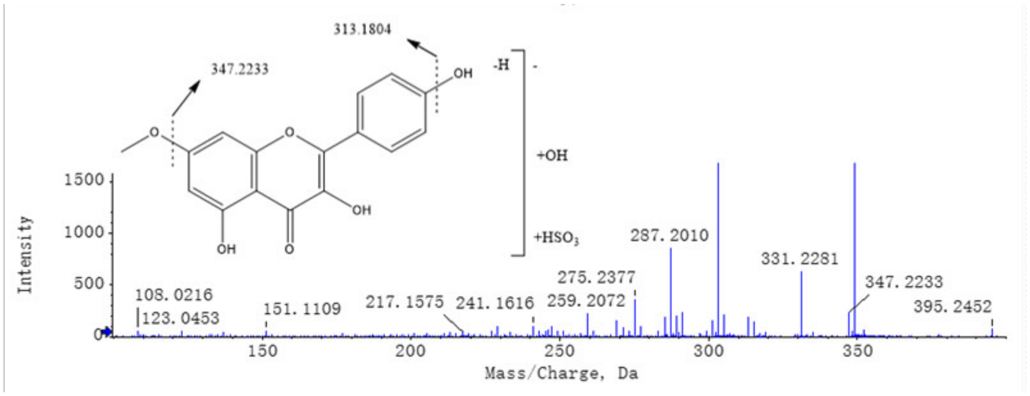

Figure S29 The negative MS/MS spectra of metabolite M29

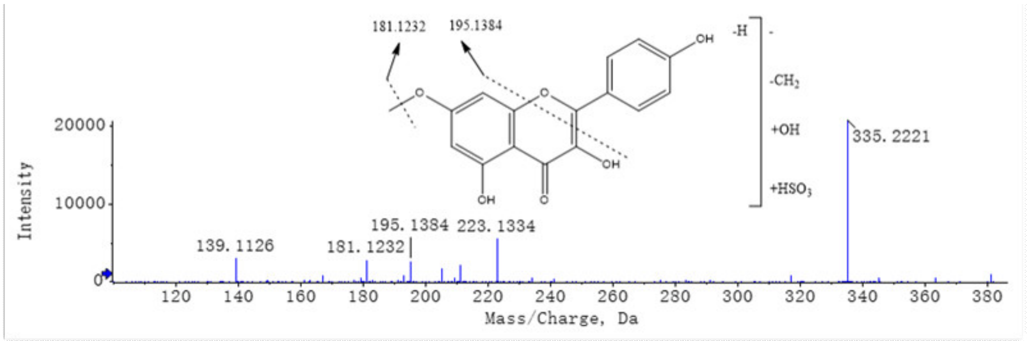

Figure S30 The negative MS/MS spectra of metabolite M30

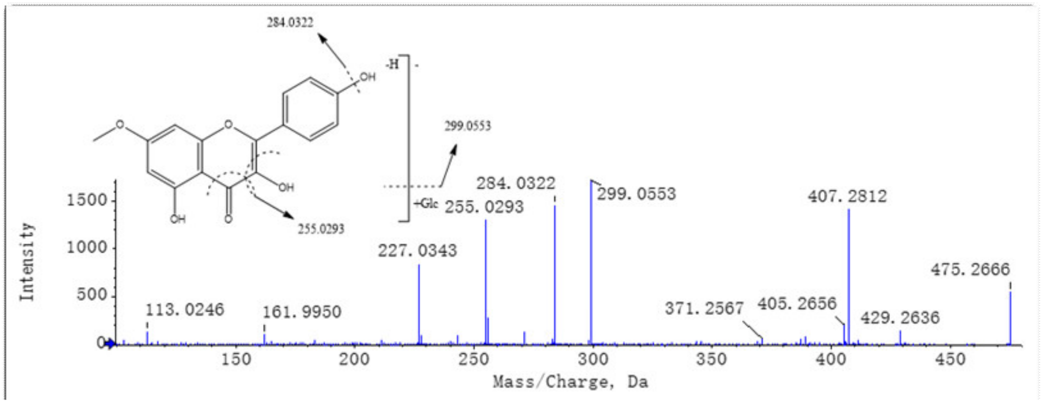

Figure S31 The negative MS/MS spectra of metabolite M31

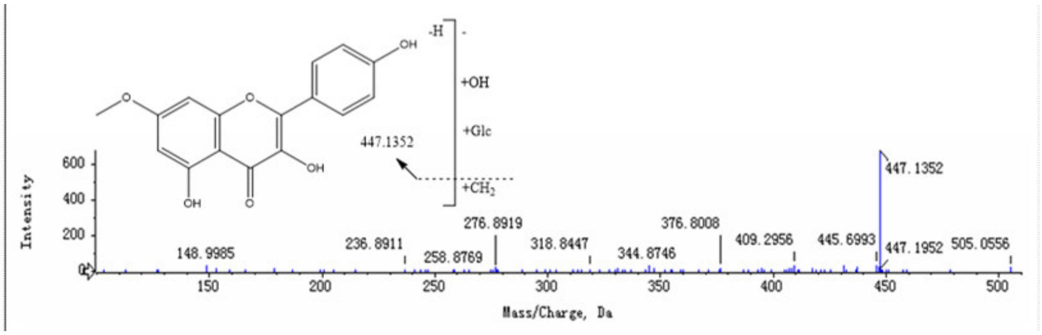

Figure S32 The negative MS/MS spectra of metabolite M32

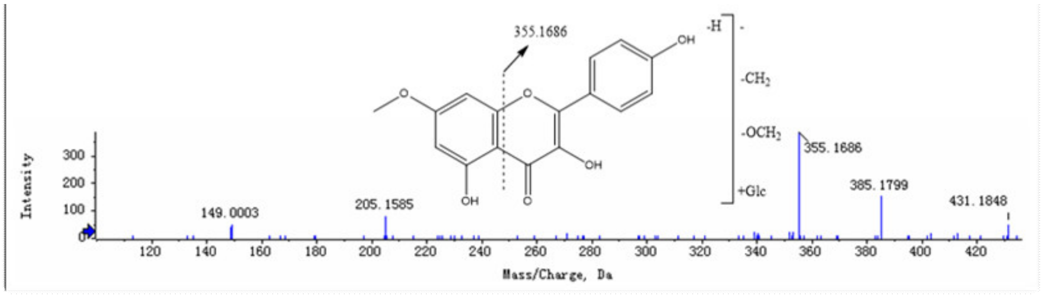

Figure S33 The negative MS/MS spectra of metabolite M33

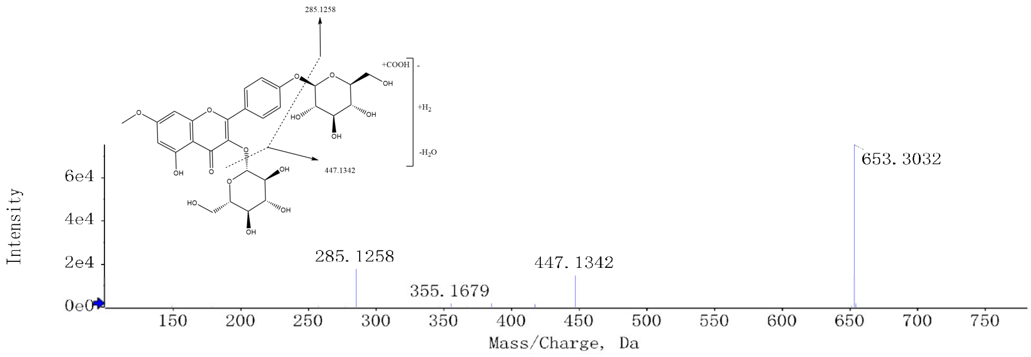

Figure S34 The negative MS/MS spectra of metabolite M34
